# Institutional trust, conspiracy beliefs and Covid-19 vaccine uptake and hesitancy among adults in Ghana

**DOI:** 10.1371/journal.pgph.0003852

**Published:** 2024-10-16

**Authors:** Meshack Achore, Joseph Asumah Braimah, Robert Kokou Dowou, Vincent Kuuire, Martin A. Ayanore, Elijah Bisung

**Affiliations:** 1 Department of Population Health, Hofstra University Hempstead, Hempstead, New York, United States of America; 2 Department of Public Health, St. Lawrence University, Canton, New York, United States of America; 3 Department of Epidemiology and Biostatistics, Fred N. Binka School of Public Health, University of Health and Allied Science, Ho, Ghana; 4 Department of Geography, Geomatics & Environment, University of Toronto Mississauga, Toronto, Canada; 5 Department of Health Policy Planning and Management, University of Health and Allied Sciences, Ho, Ghana; 6 Department of Kinesiology and Health Studies, Queens University, Kingston, Canada; University of Colorado Denver - Anschutz Medical Campus: University of Colorado - Anschutz Medical Campus, UNITED STATES OF AMERICA

## Abstract

Vaccine hesitancy is considered one of the ten threats to global health. In the context of the COVID-19 pandemic, vaccine hesitancy may undermine efforts toward controlling or preventing the disease. Nevertheless, limited research has examined vaccine hesitance, particularly in low- and middle-income countries (LMICs). It is thus imperative to examine how institutional trust and conspiracy belief in tandem influence the uptake of COVID-19 vaccines. Using data (n = 2059) from a cross-sectional study in Ghana, this study examines the association between institutional trust, conspiracy beliefs, and vaccine uptake among adults in Ghana using logistics regression. The regression model (model 3) adjusted for variables such as marital status, age, gender, employment, income, and political affiliations. The results show that individuals were significantly less likely to be vaccinated if they did not trust institutions (OR = .421, CI = .232–.531). Similarly, we found that individuals who believed in conspiracy theories surrounding the COVID-19 vaccine were less likely to be vaccinated (OR = .734, CI = .436–.867). We also found that not having a COVID-19-related symptom is associated with vaccine refusal (OR = .069, CI = .008–.618). Similarly, compared to those with a vaccine history, those without a vaccine history are less likely to accept the COVID-19 vaccine (OR = .286, CI = .108–.756). In conclusion, our results demonstrate the need for enhanced education to tackle conspiracy beliefs about the disease and enhance vaccine uptake. Given the role of trust in effecting attitudinal change, building trust and credibility among the institutions responsible for vaccinations ought to be prioritized.

## 1. Background

Although no longer a public health emergency of international concern, the coronavirus disease (COVID-19) remains a significant public health burden, affecting individuals and health systems. The outbreak of the virus was characterized by the fierce race for vaccines as part of several measures to help curb the spread of the disease. Vaccines are particularly useful in reducing transmission, hospitalizations, and infection severity. Indeed, mass vaccinations have been effective in curtailing the spread of most infectious diseases across the globe. Following their approvals for use as a COVID-19 containment measure, COVID-19 vaccines have been reported to save 14.4 billion deaths [1] with over 13 billion vaccine doses administered [2].

In line with global public health recommendations, the Government of Ghana adopted mass vaccination as one of its strategies to curtail the spread of COVID-19 and was the first country in sub-Saharan Africa (SSA) to receive COVID-19 vaccines from the COVAX facility. As of March 26, 2023, nearly 22.5 million doses of COVID-19 vaccine were administered in Ghana [2]. The effectiveness of vaccines in reducing infections, including COVID-19 however depends to a great extent on their acceptance and use. In the case of the COVID-19 pandemic, its earlier stages were characterized by widespread uncertainty, contradictory narratives, and conspiracy beliefs, largely owing to limited available data and scientific uncertainty [3]. This unique nature of the pandemic (scientific uncertainty, accelerated vaccine development) along with Ghana’s relatively weak healthcare system and general distrust in the healthcare system can affect vaccine rollout. While a national survey on vaccine acceptance in Ghana is absent, a few cross-sectional studies in Ghana point to appreciable levels of vaccine rejection and vaccine hesitance [4–6]. This could be undermining Ghana’s efforts towards containing the COVID-19 pandemic. Notwithstanding the observed benefits of vaccines, especially in reducing and preventing infections and deaths, vaccine hesitancy remains a major health concern in many countries across the world, rendering it one of the top 10 global public health concerns in 2019.

Vaccine hesitancy, referred to as “the reluctance or refusal to vaccinate despite the availability of vaccines” [7] is a complex phenomenon. Previous studies link it to distrust and conspiracy beliefs. For example, Jennings and colleagues in a nationally representative study in the UK found that both distrust and belief in conspiracy increase vaccine hesitancy [8].

Also, Bertin et al. [9] observed that conspiracy beliefs and conspiracy mentality adversely affect intentions to vaccinate against COVID-19. In the specific case of COVID-19, research on the link between trust and conspiracy beliefs and COVID-19 vaccine uptake reveals mixed results. Some of these studies conclude that trust in COVID-19 vaccines increases people’s willingness to accept them [4, 10]. On the contrary, distrust in vaccines produces doubts and a lack of confidence. Likewise, conspiracy beliefs, defined as a belief system that provides an alternative explanation to a phenomenon and asserts the existence of a conspiracy by powerful groups despite the existence of other plausible explanations [11], also influence people’s vaccine intentions during infectious disease outbreaks [8, 9]. In addition to distrust and conspiracy beliefs, studies on vaccine acceptance and or hesitancy identify various individual and sociodemographic factors as drivers of vaccine uptake. These include gender, education, and income [4, 12].

This paper expands the current knowledge on COVID-19 vaccine uptake by examining the relationship between conspiracy beliefs and trust in institutions and the willingness of adult Ghanaians to vaccinate against COVID-19. As it stands, most of the evidence on why people accept or reject the vaccine, especially in SSA is hypothetical and was assessed prior to vaccine development and widespread availability. Thus, participants’ answers to the survey questions could be affected by the fact that there were no approved vaccines. In addition, there is little evidence on how institutional trust influences COVID-19 vaccine uptake in Ghana and many other countries in SSA. The few studies that have examined this issue have mostly focused on trust alone, with little attention paid to how other factors, such as conspiracy beliefs, impact vaccine uptake. It is thus imperative to examine how trust and conspiracy belief in tandem influence the uptake of COVID-19 vaccines after approved vaccines became widely available. In the context of changing infections, beliefs about the virus, and knowledge about its mode of spread, this study will provide empirical evidence for tailored COVID-19 containment intervention programming. Understanding how trust facilitates or hinders health decision-making will provide the evidence base for policy decision-making and programming. We hypothesized that distrust in institutions and belief in a conspiracy can reduce COVID-19 vaccine uptake. Likewise, distrust in institutions and belief in conspiracies can increase vaccine hesitancy.

## 2. Materials and methods

### 2.1 Study context and setting

This study was conducted in four cities in Ghana: Wa, Tamale, Kumasi, and Accra. Ghana is generally divided into four distinct geographical regions. The North, where Wa and Tamale are located, is in the high plains, and the South, where Accra and Kumasi are located, is in the low plains. Ghana recorded its first COVID-19 case in Accra on March 12th, 2020, amidst widespread belief that the virus could not survive in Ghana’s warm, tropical climate [13]. The incidence rate of COVID-19 increased across the country, mainly in the more extensive metropolitan areas, including Accra, Kumasi, Tamale, and Wa, resulting in an increase in the country’s overall prevalence rate of COVID-19 by December 2020. The first COVID-19-related death was reported in Kumasi on March 21, 2020. By March 31, 2020, Ghana had recorded 161 cases of COVID-19 and five deaths, prompting the government to start prohibiting public meetings and closing churches, mosques, and schools, particularly in the major cities. Ultimately, the borders were closed, and travelers who made it into the country before this measure had to quarantine for 14 days [14].

On March 30, 2020, the country was completely locked down, prohibiting intercity travel. These restrictions were periodically evaluated, and changes were made accordingly. For instance, private funerals and religious gatherings were allowed, but in fewer numbers. Notwithstanding these precautions, anecdotal data shows that false information about the COVID-19 virus was circulating on several social media platforms. Prominent among these were the misinformation and misconceptions that the COVID-19 virus does not affect blacks/Africans and young people; the virus is a biological weapon targeted at Africans; the virus cannot survive in Africa’s hot climate [15, 16]. There were also conspiracy theories in circulation, including the belief that the virus would change the world order and ultimately end the world [15, 16]. Similar misinformation and conspiracy theories were made about the COVID-19 vaccine.

### 2.2 Study design and sampling technique

We employed a cross-sectional study design to collect primary data from four administrative cities in Ghana (Accra, Kumasi, Tamale, and Wa). The sampling followed a two-step process. First, we randomly selected our primary sampling units (PSUs) from a sampling frame comprising cities and enumeration areas (EAs) list. Using a systematic sampling technique, we then selected our secondary sampling units (SSUs) (i.e., households) from each selected EA. The target population was represented by adult residents over 18 years in the respective cities during the pandemic. We used Krejci and Morgan’s (1970) formula for estimating sample size based on the known population; a target sample of 2000 was sufficient at a 95% confidence level based on the cities’ respective populations. A total of 2059 respondents participated in the study.

### 2.3 Data collection

The survey was conducted using electronic tablets and a structured questionnaire. The recruitment and data for this study were collected from 25 October 2022 to 5 October 2023. The questionnaire was tested for both content validity and reliability. For content validity, we submitted the questionnaire to experts in the field, including academics and practitioners who validated the questionnaire’s content. For reliability, we initially piloted with 2% of the targeted households and assessed the reliability using Cronbach’s alpha. Our calculation returned a Cronbach’s alpha of 0.86, indicating strong reliability [17]. Four research assistants who were fluent in English and at least two regional languages (such as Twi, Ga, and Dagbani) to administer the survey. The questionnaire for the survey was created in English and then translated into the local languages and translated back into English. In line with research conducted during active Ebola epidemics [18], the survey gathered information on exposure to COVID-19, knowledge of behavioral changes brought on by COVID-19, trust in COVID-19 response measures, institutional trust, demographics, and socioeconomics variables, and health-seeking behaviors of participants.

### 2.4 Measures

The study examined two dependent variables (vaccine uptake and vaccine hesitancy) and several independent variables, such as education level, employment status, gender, age, and marital status. Details of how these variables were measured are described below.

#### Vaccine uptake and hesitancy

Vaccine uptake was measured by asking participants if they had taken at least one dose of the COVID-19 vaccine. Participants answered Yes or No. Based on the response, we created a binary variable with 1 denoting vaccine uptake and 2 denoting vaccine hesitancy. The vaccine hesitance served as a second dependent variable. It was measured by asking those who answered No to the vaccine uptake question if they would be willing to get vaccinated. Participants indicated Yes or No in response to the question. We then dichotomized the variable, with 1 denoting willingness to be vaccinated and 2 denoting complete refusals.

#### Institutional trust

Adapted from Sato’s [19] Afrobarometer survey, trust was measured by asking participants how much they trust each of the following institutions: the president or presidency, the parliament, the ruling political party, traditional leaders, religious leaders, health administrators, and health workers (e.g., doctors, nurses, midwives). Using a Likert scale ranging from 1 = not at all to 4 = a lot, participants responded to the above questions by selecting one option. The responses were split into two groups, with participants selecting 1 and 2, categorized as those who lack trust, and those selecting 3 and 4, denoting institutional trust.

#### Conspiracy beliefs

These were measured by asking participants to either agree or disagree with the following statements: A hidden organization is behind the spread of the coronavirus; pharmaceutical companies are behind the spread of the coronavirus; financial interests lie behind the spread of the coronavirus; and the coronavirus pandemic is made up. The responses were yes and no. Participants who agreed with at least one of the statements were classified as those with conspiracy beliefs and coded (1), and those who disagreed with all the statements were considered those without conspiracy beliefs and coded 2. Believers and nonbelievers were further classified as 1 (distrust) and 2 (trust) respectively.

#### Other variables

We adjusted for several socio-demographic variables such as level of education, marital status, employment status, age, and health insurance status as demonstrated in previous literature [6, 20, 21]. In addition, we controlled for whether someone has willingly received any vaccine in the past and has experienced COVID-19-related symptoms. We assumed these variables would impact one’s willingness or hesitancy to receive a vaccine.

## 3. Data analysis

The Statistical Package for Social Sciences (SPSS) program was used for the statistical analysis (version 25.0). Using frequencies and percentages for the categorical data, descriptive analysis was conducted for the sociodemographic factors (Sex, educational status, occupation, region of residence, marital status, and religion). Means and standard deviations were employed for continuous variables. Logistics regression models were used for inferential statistical analysis. (1) COVID-19 vaccine uptake (vaccine acceptance = 1; non-vaccinated = 2), and (2) COVID-19 vaccine hesitance (willingness to vaccinate = 1; complete refusals = 2) were the main outcome variables of interest. The models generated are predictive in nature. Before fitting the regression models, the multicollinearity of all relevant explanatory variables was checked. We generated six vaccine acceptance and hesitance predictor models using bivariate and multivariate logistic regressions. The first model (model 1) examined the relationship between vaccine acceptance and trust. Model 2 examines the relationship between vaccine acceptance and conspiracy beliefs. Model 3 examined the relationship between vaccine acceptance, trust, and conspiracy beliefs while adjusting for socioeconomic variables (i.e., age, sex, employment status, marital status, money, etc.). We further explored vaccine hesitancy to see if those hesitant are willing to receive the vaccine in the future using models 4, 5, and 6. Model 4 examined the relationship between vaccine hesitancy and trust. Model 5 examines the relationship between vaccine hesitancy and conspiracy beliefs. In model 6, we added socioeconomic variables. The results are presented as odds ratios (OR), and the statistical significance is assessed using 95% confidence intervals (CI).

### 3.1 Ethics approval

The Queen’s University General Research Ethics Board (GREB) has reviewed and approved this study. A written informed consent had been obtained from participants before including them in the study. The purpose of the study was also explained to them before interviewing them. They were informed of their right to stop at any point in time of the interview process when they feel so, and also assured that any statement or comments including that of privacy will remain protected. The confidentiality of the participants was adhered to by ensuring that no information from participants was disclosed to any third party. The participants were recognized by codes and numbers instead of their real names.

## 4. Results

Table 1 displays the participants’ demographics. Among those who reported receiving at least one dose of the COVID-19 vaccine, 35.3% are women and 64.7% are men. Of the non-vaccine individuals, 44.5% were men and 55.5% were women. A majority (53.1%) of the participants who refused the vaccine were employed. The majority (53.4%) of those aged 18 to 30 were non-vaccine; of this proportion, 52.5% refused the vaccine. Most complete vaccine refusers (98.2%) reported that they do not have trust in the expert community. In addition, a high majority of participants who believed in conspiracy theories surrounding the vaccine refused it (80.6%).

**Table 1:** Participants demographics.

Variables		Vaccine uptake		Vaccine Hesitant	
		Vaccinated (n = 947)	Non-Vaccine (n = 1096)	Willingness to vaccinate(n = 157)	Complete refusals (n = 939)
Sex	Male	333(35.3%)	476(44.5%)	59(37.6%)	417(44.4%)
	Female	610 (64.7%)	594(55.5%)	98(62.4%)	496(55.6%)
Employment status	Employed	524(55.3%)	578(52.7%)	79(50.3%)	499(53.1%)
	Unemployed	423(44.7%)	518(47.3%)	78(49.7%)	440(46.9%)
Age	18-30yrs	402(42.4%)	585(53.4%)	92(58.6%)	493(52.5%)
	31-40yrs	237(25.1%)	265(24.2%)	39(24.8%)	226(24.1%)
	>40yrs	308(32.5%)	246(22.4%)	26(16.6%)	220(23.4%)
Average monthly income	<GHC2000	657(69.4%)	713(65.1%)	110(70.1%)	603(64.2%)
	Ghc2000- Ghc 5000	121(12.8%)	120(10.9%)	11(7.0%)	109(11.6%)
	> Ghc5000	169(17.8%)	263(24%)	36(22.9%)	227(24.2%)
Political affiliations	Current government	248(26.2%)	302(27.6%)	55(35%)	247(26.3%)
	Opposition	290(30.6%)	365(33.3%)	50(31.8%)	315(33.5%)
	None	409(43.2%)	429(39.1%)	52(33.2%)	337(38.2%)
Religion	Christian	440(46.7%)	645(59.2%)	79(50.3%)	566(60.7%)
	Muslim	498(52.8%)	427(39.2%)	75(47.8%)	352(37.4%)
	African Traditionalist	5(0.5%)	18(1.6%)	3(1.9%)	15(1.9%)
Marital Status	Single	242(25.7%)	415(38.1%)	60(38.2%)	355(37.8%)
	Married	549(58.3%)	673(61.9%)	97(61.8%)	576(61.9%)
Education	No formal education	204(21.7%)	199(18.2%)	27(17.2%)	172(19.3%)
	Formal education	743(78.3%)	887(81.8%)	130(82.8%)	757(80.7%)
Study city	Accra	160 (16.9%)	354(32.4%)	14(8.9%)	340(36.4%)
	Kumasi	190 (20.1%)	297(27.2%)	54(34.4%)	243(25.9%)
	Tamale	214(22.6%)	301(27.6%)	50(31.8%)	251(26.9%)
	Wa	379(40.4%)	140(12.8%)	39(24.9%)	101(10.8%)
Covid-19 status	Positive	63(7.1%)	30(2.7%)	5(3.2%)	25(2.7%)
	Negative	880(92.9%)	1066(97.3%)	150(96.8%)	916 (97.3%)
Willingly take any vaccination	Yes	441(47.1%)	183(16.7%)	44(28%)	139(14.8%)
	No	497(52.9%)	839(76.8%)	111(70.7%)	728(77.9%)
	Prefer not to say	6 (0.6%)	71(6.5%)	2(1.3%)	69(7.3%)
Health insurance status	Valid health insurance	641(68.4%)	638(58.1%)	114(72.6%)	524(56.8%)
	No health insurance	296(31.6%)	442(40.9%)	43(27.4%)	399(43.2%)
Self-rated health	Excellent	145(15.4%)	1030(94.1%)	148(94.3%)	882(94%)
	Poor	27(2.9%)	64(5.9%)	9(5.7%)	55(6%)
Access to COVID-19 media campaigns	Very often	504(53.3%)	735(67.4%)	114(72.6%)	621(66.6%)
	Never	30 (3.2%)	357(32.6%)	43(27.4%)	314(33.4%)
Conspiracy beliefs	Nonbelievers	310(32.7%)	200(18.2%)	52(33.1%)	148(15.8%)
	Believers	637(67.3%)	896(81.8%)	105(66.9%)	757(80.6%)
Trust	Trust	25(2.6%)	20(1.8%)	3(1.9%)	17(1.8%)
	Distrust	922(97.4%)	1076(98.2%)	154(98.1%)	922(98.2%)

Table 2 below presents bivariate and multivariate logistic regressions of institutional trust and conspiracy beliefs in COVID-19 and vaccine uptake in Ghana. It is important to note that vaccine hesitancy and “less likely to receive a vaccine” are used interchangeably in this section. Models 1 and 2 display the result of bivariate logistic regression. Model 1 shows that individuals were significantly less likely to be vaccinated if they did not trust institutions *(OR =* .*421*, *CI =* .*232–*.*531)*. Similarly, model 2 indicates that individuals who believed in conspiracy theories surrounding the COVID-19 vaccine were less likely to be vaccinated *(OR =* .*734*, *CI =* .*436–*.*867)*. Model 3 presents results from a multivariate logistic regression. Overall, the odds of being vaccinated further decreased due to distrust *(OR =* .*218; CI =* .*111–*.*429)* and conspiracy beliefs *(OR =* .*33; CI =* .*191–*.*572)* after adjusting for a range of socioeconomic and demographic variables. We found that being associated with political parties other than the two major political parties (the New Patriotic Party and the National Democratic Congress), age, education, and city were associated with higher odds of being vaccinated. Those who belonged to other political parties were 1.3 times more likely to be vaccinated *(OR = 1*.*382; CI = 1*.*029–1*.*856)*. Conversely, those who tested positive for COVID-19 .164 (*CI =* .*045-*.*601)* were less likely to get vaccinated.

**Table 2:** Bivariate and multivariate logistic regression of institutional trust and conspiracy beliefs in COVID‑19 and vaccine uptake in Ghana.

		Model 1: Association between vaccine uptake and trust	Model 2: Association between vaccine uptake and conspiration beliefs	Model 3: Adjusted association between vaccine uptake, trust and conspiration beliefs
		OR(CI)	OR(CI)	aOR(CI)
**Institutional trust**	Trust	Ref		Ref
	Distrust	.421(.232-.531) *		218(.111-.429) **
**Conspiracy beliefs**	Non-believers		Ref	Ref
	believers		.734(.436-.867) *	.330(.191-.572)**
**Sex**	Female			Ref
	Male			1.328(1.034–1.706)
**Employment Status**	Unemployed			Ref
	Employed			.946(0.711–1.258)
**Income**	<GHC5000			Ref
	Ghc5001-Ghc 10,000			.862(0.604–1.231)
	> Ghc10,000			.738(0.456–1.196)
**Political affiliation**	Current government			Ref
	Leading opposition party			1.070(.780–1.468)
	Others			1.382 (1.029–1.856)**
**Age**	18-30yrs			Ref
	31 – 40yrs			2.248(1.532–3.299) **
	>40yrs			1.653(1.178–2.318)**
**Marital Status**	single			Ref
	Married			.746(.357–1.560)
**Religion**	Christian			Ref
	Muslim			1.819(.310–10.681)
	African Traditionalist			2.420(.399–14.688)
**Education**	No formal education			Ref
	Formal education			1.427(1.010–2.016)**
**City**	Accra			Ref
	Kumasi			7.966(4.883–12.995) *
	Tamale			9.267(5.443–15.780) *
	Wa			6.723(4.274–10.575) *
**Insurance Status**	Not insured			Ref
	Insured			.936(.727–1.205)
**Have you ever tested positive to COVID-19**	Yes			Ref
	No			.164(.045-.601)**
**Have you had COVID-19-related symptoms in the past?**	Yes			Ref
	No			.573(.217–1.510)
**Have you had any past vaccinations?**	Yes			Ref
	No			.313(.099-.994)**
**Constant**				3.579

Note: OR odds ratio; 95% confidence intervals in brackets; model 3 adjusted for covariates

* p < 0.05

** p < 0.01

*** p < 0.001.

Table 3 below presents bivariate and multivariate logistic regressions of institutional trust and conspiracy beliefs in COVID-19 and vaccine hesitancy in Ghana. Models 4 and 5 display the result of bivariate logistic regression. Model 4 shows that individuals completely refused the vaccine if they did not trust institutions (OR = .987, CI = .915–1.000). Model 5 indicates that individuals who believed in conspiracy theories surrounding the COVID-19 vaccine refused the vaccine (OR = .487, CI = .285–.832) compared to their counterparts. Model 5 (multivariate results) indicates that after adjusting for several independent variables, distrust was no longer a significant predictor of the odds of vaccination. Conspiracy beliefs, however, remain significant (OR = .303, CI = .109–.841). Regarding geographical location, the unvaccinated individuals in Kumasi were willing to be vaccinated (OR = 9.533, CI = 3.356–27.080). Similar results were found with those in Tamale (OR = 2.580, CI = 1.032–1.448).

**Table 3:** Bivariate and multivariate logistic regression of institutional trust and conspiracy beliefs in COVID‑19 and vaccine hesitancy in Ghana.

		Model 4: Association between vaccine hesitancy and trust	Model 5: Association between vaccine hesitancy and conspiration beliefs	Model 6: Adjusted association between vaccine hesitancy, trust and conspiration beliefs
		OR(CI)	OR(CI)	aOR(CI)
**Institutional Trust**	Trust	Ref		Ref
	Distrust	.987(.915–1.000)*		.960(.915–1.007)
**Conspiracy beliefs**	Non-believers		Ref	Ref
	believers		.487(.285-.832) *	.303(.109-.841) *
**Sex**	Female			Ref
	Male			1.257(.786–2.011)
**Employment Status**	Unemployed			Ref
	Employed			1.523(.863–2.687)
**Income**	<GHC2000			Ref
	Ghc2000- Ghc 5,000			1.099(.549–2.198)
	> Ghc5000			1.630(.571–4.649)
**Political affiliation**	Current government			Ref
	Leading opposition party			.661(.365–1.196)
	Others			1.014(.562–1.828)
**Age**	18-30yrs			Ref
	31 – 40yrs			.549(.264–1.144)
	>40yrs			.537(.264–1.092)
**Marital Status**	single			Ref
	Married			2.295(.557–9.466)
**Religion**	Christian			Ref
	Muslim			1.290(.114–14.574)
	African Traditionalist			2.423(.197–29.746)
**Education**	No formal education			Ref
	Formal education			.718(.314–1.642)
**City**	Accra			Ref
	Kumasi			9.533(3.356–27.080)*
	Tamale			2.580(1.032–6.448) *
	Wa			1.605(.658–3.916)
**Insurance Status**	Not insured			Ref
	Insured			.717(.443–1.160)
**Have you ever tested positive for COVID-19**	No			Ref
	Yes			.309(.023–4.072)
**Have you had COVID-19-related symptoms in the past?**	Yes			Ref
	No			.069(.008-.618) *
**Have you had any past vaccinations?**	Yes			Ref
	No			.286(.108-.756) *
**Constant**				121.552

Note: OR odds ratio; 95% confidence intervals in brackets; model 6 adjusted for covariates

* p < 0.05

** p < 0.01

*** p < 0.001.

## 5. Discussion

In the context of the coronavirus pandemic, several vaccines were speedily developed as part of strategies to mitigate its spread, reduce hospitalizations, and reduce mortality. While the benefits of these vaccines have been recorded, the evidence available points to high levels of vaccine refusal and vaccine hesitancy in Ghana [6, 10] and several other contexts [4, 22]. This study contributes to the current vaccine literature by examining how trust in institutions and belief in conspiracies influence COVID-19 vaccine uptake and hesitancy among adult Ghanaians. This is particularly critical considering the evolving nature of the various.

We found institutional distrust to be significantly related to COVID-19 vaccine uptake, with individuals who distrust institutions statistically significantly more likely to be hesitant or reject the vaccine. This finding is consistent with existing vaccination literature, particularly studies pointing to the adverse effects of distrust on health behaviors, including vaccine acceptance [4, 8, 23]. For instance, in southern Ethiopia, at-risk populations that perceived COVID-19 infections and deaths reported by the government as factual were more willing to get vaccinated [24]. The authors further found a lack of trust in the effectiveness of the vaccines to induce hesitancy [24]. A recent scoping review on vaccine hesitancy in Africa by Ackah and colleagues reported a lack of trust in pharmaceutical companies causes vaccine hesitancy [4]. An earlier review of the literature on attitudes to vaccination identified distrust of doctors, government sources, and pharmaceutical companies as grounds for vaccine hesitancy [25]. During the COVID-19 pandemic, widespread scientific uncertainty, misinformation, and disinformation regarding its cause, risks and prevention prevailed [26]. These uncertainties and misconceptions, among others, sparked fears about its mitigation measures, including the safety and side effects of available COVID-19 vaccines [23, 24]. Individuals had to rely on scientific and other institutions such as governments, health systems and pharmaceutical companies for information on the disease and the subsequent vaccine rollouts. Thus, the relationships between these individuals and the institutions, viewed as the knowledge brokers, particularly their levels of trust, have the potential to influence their attitudes to vaccines, including taking the vaccines. Moreover, the lack of trust in these institutions may also provide breeding grounds for misinformation from other non-credible sources, which can negatively affect vaccine uptake [8].

Besides institutional trust, our findings suggest that beliefs in conspiracy influence vaccine hesitancy. This finding aligns with previous studies in other settings [9, 24, 27]. For example, Zewude and Habtegiorgis [24] examined at-risk populations’ willingness to take the COVID-19 vaccine in southern Ethiopia and concluded that individuals who had doubts about the existence of the disease in their area were unwilling to receive the vaccine. Several conspiracy theories and unsubstantiated beliefs emerged following the declaration of COVID-19 as a pandemic, many of which negatively affected behaviors. For instance, during its earlier stages, a section of Ugandan men considered COVID-19 a disease of “the Whites” [28]. In Ghana, the vaccine was perceived by some people to cause impotence, barrenness, or conspiracy by “whites” to kill Africans [5]. These conspiracy theories have the potential to induce distrust, non-adherence to safety guidelines, lack of control, and feelings of uncertainty and anxiety, which can discourage individuals from taking the vaccine or render them hesitant [27, 29].

Given that vaccine hesitancy or rejection may change as the pandemic evolves or new evidence emerges [24], we further examined if institutional trust and beliefs in conspiracy theories surrounding the vaccines can lead to the complete refusal of the vaccine (or willingness to take it in future). This analysis is particularly germane as vaccine hesitance may not necessarily lead to complete rejection. We found at the bivariable level that institutional trust influences willingness to take the vaccine, with individuals completely refusing the vaccine once they distrust institutions. After adjusting for relevant demographic, socioeconomic and behavioral variables, however, the association became insignificant. However, the relationship may be mediated by participants’ experiences of COVID-19-related symptoms and vaccination history. Specifically, we observed that individuals who never experienced COVID-19-related symptoms are more likely to completely refuse to take the vaccine compared with their counterparts who did. This is unsurprising given that experiencing symptoms could induce fear and uncertainty which can compel people to act. Similarly, there is evidence that having had vaccinations in the past mediated the relationship between institutional trust and willingness to take to COVID-19 vaccine [30]. In China, Wang and colleagues observed that being vaccinated against influenza in the past increased the likelihood of accepting covid-19 vaccine [23]. It is plausible that individuals who have not had any vaccines in the past are generally vaccine-hesitant and hence uninterested in the COVID-19 vaccine.

Furthermore, we observed that beliefs in the conspiracy are related to participants’ willingness to accept the COVID-19 vaccine. Specifically, we found that individuals who believe in conspiracy are more likely to completely refuse to take the vaccine compared to their counterparts who do not believe in conspiracy. There are several plausible reasons why individuals who believe in conspiracy theories will completely refuse to take the vaccine, including the feeling of distrust in institutions, fears about vaccine safety and side effects, and concerns about their efficacy [8, 25].

Although COVID-19 is no longer a global emergency of concern, our findings have several policy and research implications. Firstly, the results demonstrate the need for enhanced education to expel conspiracy beliefs about infectious diseases to enhance vaccine uptake. Given the role of trust in effecting attitudinal change, building trust and credibility of the institutions responsible for vaccinations ought to be prioritized. This can be done in several ways. For instance, building and restoring institutional trust should be incorporated into public health programs and models. Also, given that healthcare professionals are the most influential and first point of contact relative to vaccinations, they should be well informed, knowledgeable about the vaccines, and encouraged to spend more time with patients, as these can help improve their confidence. With the influence of social media in information dissemination, institutions involved in vaccinations should increase their social media presence and roll out credibility or trust-building programs. In recognition of the connection between vaccine history and the uptake of the COVID-19 vaccine, we also recommend enhanced efforts by public health professionals to encourage people to take other vaccines whose safety and efficacies have already been established, as that may position them to accept future vaccines [5].

### 5.1 Strengths and limitations

This study has some strengths and limitations worth noting. A key strength of the study is that it is one of the few studies in Ghana, a context characterized by high vaccine apathy, to employ a nationally representative sample in urban areas to examine how institutional trust and conspiracy beliefs influence COVID-19 vaccine uptake. The study highlights key policy and program areas for interventions to improve the COVID-19 vaccine and future vaccine uptake. In terms of limitations, we employed a cross-sectional design, which limits the interpretation of the findings to statistical associations between our dependent and independent variables. Also, our analysis did not account for the perceived risk of COVID-19, which has been suggested to influence health behaviors, including vaccine utilization in various contexts [31]. Lastly, given the cross-section nature of the study, we could not make causal inferences.

## 6. Conclusions

This study examined the association between institutional trust, conspiracy beliefs, and COVID-19 vaccine uptake among adult Ghanaians. Overall, the findings reveal that distrust in institutions and belief in conspiracy theories shape attitudes toward vaccines as they induce hesitancy and/or refusal of COVID-19 vaccines. In the context of this, there is the need for a clear public health agenda, particularly relating to the control of infectious diseases to also focus on building institutional trust and tackling conspiracy beliefs. Moreover, qualitative research into how distrust in institutions and beliefs in conspiracy theories are (re)constructed during pandemics and how they shape preventive behaviours including vaccine uptake will help provide context to our quantitative findings and directions for public health program intervention.

## Supporting information

S1 Checklist Inclusivity in global research. (PDF)
