# Supplementary material for: Institutional trust, conspiracy beliefs and Covid-19 vaccine uptake and hesitancy among adults in Ghana
Source: PLOS Glob Public Health. 2024 Oct 16;4(10):e0003852. doi: 10.1371/journal.pgph.0003852 (PMC11482693; doi:10.1371/journal.pgph.0003852)
Supplement: Attachment — Submitted filename: response_COVID.pdf [file pgph.0003852.s004.pdf]

Response to reviewers

Reviewer #2: Comment to the authors on "Institutional Trust, Conspiracy Beliefs and Covid-19 Vaccine Uptake and Hesitancy among Adults in Ghana"

Background

While an attempt has been made to justify the need for this important study in light of COVID-19 public health concerns, there is still much to be done. For instance, a cursory search revealed that the following study “Morgan, A. K., Aziire, M. A., Cobbold, J., Agbobada, A. A., & Kudzawu, S. K. (2023). Hesitant or not: A cross-sectional study of socio-demographics, conspiracy theories, trust in public health information, social capital and vaccine hesitancy among older adults in Ghana. Human Vaccines & Immunotherapeutics, 19(1), 2211495” was excluded from the literature relied on. Despite the close proximity, not only in geographical or conceptual scope,. I believe a thorough literature search is needed to ground the premise of the study and also for the discussion of the findings thereof.

We thank the reviewer for this comment. We believe the relevant literature used in the study was considered and duly cited in the manuscript. However, we have reviewed the literature again to see if there are other articles, including the one suggested by the reviewer, that could strengthen our arguments and incorporate them into the manuscript. We added four more studies (Baffoe & Nortey, 2021; Brackstone et al., 2022; Kowalska-Duplaga, & Duplaga, 2023; Morgan et al., 2023) to the background.

The sentence reads: This paper expands the current literature on COVID-19 vaccine uptake (Baffoe & Nortey, 2021; Brackstone, et al., 2022; Kowalska-Duplaga, & Duplaga, 2023; Morgan et al., 2023) by examining the relationship between conspiracy beliefs and trust in institutions and the willingness of adult Ghanaians to vaccinate against COVID-19. (See page 6, lines 113-116)

Study Context and Setting

The study context rather looks outdated. Spruce it up to make it current, particularly with regard to the figures, etc...

We thank the reviewer for this comment. We are unsure what the reviewer means by outdated. Our description of the study context focused on how COVID-19 evolved in Ghana, particularly how it started and the false information circulating on social media that impacted vaccine uptake, hence the need for this study. The goal is to provide a historical context to COVID-19, which, in our view, helps situate our findings.

Results

Use contingency tables and chi square texts to show if there’s any form or association between vaccine uptake or otherwise and the socio-demographic factors in Table 1.

We thank the reviewer for this comment. We decided to present our findings this way to avoid repetition since we tested the relationships/associations in Tables 2 and 3 using logistic regressions.

Discussion

What are the factors that account for the low vaccine uptake among those who tested positive for COVID-19?

We thank the reviewer for this comment. Several factors might have accounted for this, including perceived immunity, vaccine safety concerns, and misinformation. The following paragraph was added:

We also found that those who tested positive for COVID-19 were less likely to accept the vaccine. Several factors might have accounted for this finding. Individuals who have recovered from COVID-19 frequently assert that natural immunity provides adequate protection against subsequent infections, resulting in a diminished propensity to pursue vaccination. This perception is not entirely unfounded; a study published by Hall et al. (2021) indicated that the risk of reinfection was markedly reduced for at least six months following the initial infection, leading to the belief that vaccination may be unnecessary. Moreover, safety apprehensions substantially affect vaccine acceptance, especially among individuals who have previously contracted COVID-19. Individuals apprehend possible side effects or contend that vaccination may intensify any lingering symptoms from their prior infection (Freeman et al., 2021; Krammer et al., 2021). The apprehension regarding these adverse reactions may deter individuals from receiving vaccinations. The proliferation of misinformation and conspiracies regarding COVID-19 vaccines has substantially impacted vaccine acceptance, especially among individuals who have previously tested positive for the virus. Research indicates that misinformation can modify individuals' risk perceptions and diminish trust in the safety and effectiveness of vaccines (Loomba et al., 2021). This misinformation frequently exacerbates skepticism regarding vaccination. Addressing misinformation via focused educational initiatives and clear communication is essential to enhance vaccine acceptance in this demographic. (See pages 19-20, lines 398-415).

How did the definition and categorization of the variables influence the results or findings?

We appreciate this question. We know how categorizing variables influences the results; our study is no exception. The influence can only be ascertained after trying different categorizations, which we did not do. Our categorization is in line with the literature.

Reviewer #4: This is an interesting study which focuses on potential impediments to vaccine uptake in low and middle income countries, including issues of institutional trust and potential conspiracy theories. I am reviewing for the first time a revised version of the document. Previous reviewers have focused primarily on statistical methodologies and I feel that all of these comments have been adequately addressed by the authors. The manuscript is nearly ready for publication but would benefit from selected revisions that are noted below under "SPECIFIC RECOMMENDATIONS"

Currently there are numerous published studies that assess levels and predictors of vaccine hesitancy surrounding the COVID=19 pandemic. Many of these papers express the need for expansion of the role that public health staff and institutions should play to further reduce vaccine hesitancy. However, there are often no specific recommendations as to what courses of action that the public health sector could play and what unique strengths and weaknesses do they bring.

This paper in the Discussion section (Lines 390 to 404) does provide some specific recommendations. However it would be extremely helpful it they could provide relevant references for each of their recommendations. Specifically, on how to build trust in public institutions, increasing provider's capacity and ability to provide patient education, how to better use social media, and expanding information to patients on a wider range of vaccines.

We thank the reviewer for the comment. A few references have been added to the recommendations. (See page 20, lines 416-430)

SPECIFIC RECOMMENDATIONS: (I am using the revised manuscript and the accompanying Line numbering)

ABSTRACT:
Line 34: I would suggest revising the sentence, "Vaccine hesitancy is considered by the World Health Organization as one of the ten threats to global health. "

We thank the reviewer for the comment. This has been addressed. We modified the text to read:
Vaccine hesitancy poses a significant threat to efforts to control or prevent the spread of the COVID-19 virus. Despite this, there remains a notable gap in research examining vaccine hesitance, particularly among adults in low—and middle-income countries (LMICs). Consequently, it is imperative to investigate how institutional trust and conspiracy beliefs influence the uptake of COVID-19 vaccines in tandem. (see page 2, lines 33-38)

Line 36: The authors use the terms, "vaccine hesitancy" and "vaccine hesitance" interchangeably. Is there a distinction between these two terms??

We thank the reviewer for the comment. The text has been modified to read:

Vaccine hesitancy poses a significant threat to efforts to control or prevent the spread of the COVID-19 virus. Despite this, there remains a notable gap in research examining vaccine hesitance, particularly among adults in low—and middle-income countries (LMICs). Consequently, it is imperative to investigate how institutional trust and conspiracy beliefs influence the uptake of COVID-19 vaccines in tandem. (see page 2, lines 33-38)

MAIN MANUSCRIPT:
Line 64-65: While I agree that COVID-19 is no longer officially considered a public health emergency of international concern, it remains a public health priority for WHO.
We agree entirely; hence, that statement has been deleted.

Line 111. Would suggest writing out in full.." Sub Saharan Africa"

Done as suggested by the reviewer

Lines 325-326. This sentence is incomplete. "This is particularly critical considering the evolving nature of the various........???????" Please complete the sentence.

We thank the reviewer for spotting this. We meant virus, but it autocorrected. This has been modified to read:

This is particularly critical considering the evolving nature of the virus.

Reviewer #5: The following need to be incorporated before final approval of the manuscript:
1. Line 71 - the 14.4 billion should be replaced by 14.4 million

We thank the reviewer for the comment. This has been corrected.

2. Line 77-78 : Acceptance / use - need to add the dimension of effective quality chain and concerns about the quality at least in the description for the COVID vaccine, which had very strict temparature requirments - that was not easy to maintain tropical Ghana.

We thank the reviewer for this comment. The sentence below has been added:

The problem is further compounded by worries about the COVID-19 vaccine's quality, which include the efficiency of the supply chain and the perceived integrity of the distribution procedures (GAVI, 2021). (See page 3, lines 79-82)

3. The authoris need to make a specific comment upfront about "Institutional Trust" as represented by the comments on people in the instituitons. For example, the authors did not ask, do you trust the health centre or do your trust dispensary or hospital. Potentially, there is a difference between institutions, as opposed to individuals in the institutions. It is best to qualify it upfront both in the Methods and in the discussion sections.

We thank the reviewer for this comment. This sentence paragraph has been added:
Institutional entities herein refer to influential individuals and groups that wield power and influence within a community. These include leaders from traditional and religious backgrounds who shape social norms; politicians and governance institutions like the president, the parliament, and the ruling political party; and health-related authorities like doctors, nurses, and midwives, who are essential to the healthcare system
