# Supplementary material for: Institutional trust, conspiracy beliefs and Covid-19 vaccine uptake and hesitancy among adults in Ghana
Source: PLOS Glob Public Health. 2024 Oct 16;4(10):e0003852. doi: 10.1371/journal.pgph.0003852 (PMC11482693; doi:10.1371/journal.pgph.0003852)
Supplement: Attachment — Submitted filename: Response to Editors.docx [file pgph.0003852.s002.docx]

Response to Editors

1. Before we can proceed, please report in the Methods section the day, month and year of the start and end of the recruitment period for this study. Please note that if this information is not included when your manuscript is resubmitted, it may be rejected.

**Response: Thank you very much. In the revised manuscript, we have provided the day, month and year of the start and end of the recruitment period for this study (see page 6)**

2. Also, we kindly ask that you address the discrepancy in the ethics documentation. The individual Dr. Ebenezer Dassah named as an investigator on the ethics documents is not an author on the present submission. As we would generally expect the investigator named on the ethical approval to be an author on the submission, can you please explain this situation?

**Response: Thank you very much. Dr. Ebenezer Dassah did not contribute to the current study, though he was part of the general project. There are other parts of the bigger project he contributed to, and will be the author of those papers.**
