# Supplementary material for: Institutional trust, conspiracy beliefs and Covid-19 vaccine uptake and hesitancy among adults in Ghana
Source: PLOS Glob Public Health. 2024 Oct 16;4(10):e0003852. doi: 10.1371/journal.pgph.0003852 (PMC11482693; doi:10.1371/journal.pgph.0003852)
Supplement: Attachment — Submitted filename: Response to Editor.docx [file pgph.0003852.s003.docx]

**Response to Editor**

1. Please amend your detailed Financial Disclosure statement. This is published with the article. It must therefore be completed in full sentences and contain the exact wording you wish to be published.

a. State the initials, alongside each funding source, of each author to receive each grant.

b. State what role the funders took in the study. If the funders had no role in your study, please state: “The funders had no role in study design, data collection and analysis, decision to publish, or preparation of the manuscript.”

c. If any authors received a salary from any of your funders, please state which authors and which funders.

If you did not receive any funding for this study, please simply state: “The authors received no specific funding for this work.”

**Response: The financial disclosure has been amended to include all your recommendations**

2. Inclusivity-in-global-research-questionnaire.docx is currently uploaded as file type “Other”, which is not viewable by the reviewers. Please change the file type(s) to 'Supporting Information' and include a legend in the manuscript if you wish it/them to be included in review.

**Response: The file type for Inclusivity-in-global-research-questionnaire.docx has been modified into “supporting information”**

3. We have noticed that you have uploaded Supporting Information files, but you have not included a list of legends. Please add a full list of legends for your Supporting Information files after the references list.

**Response: the supporting information has been modified. The file has been renamed appropriately**
