# Supplementary material for: Institutional trust, conspiracy beliefs and Covid-19 vaccine uptake and hesitancy among adults in Ghana
Source: PLOS Glob Public Health. 2024 Oct 16;4(10):e0003852. doi: 10.1371/journal.pgph.0003852 (PMC11482693; doi:10.1371/journal.pgph.0003852)
Supplement: Peer review history

# Author comment: Author response to previous submission

**DOI:** 10.1371/journal.pgph.0003852.r001

25 Jan 2024

Attachment Submitted filename: Response to Editors.docx

# Peer review: Decision Letter 0

**DOI:** 10.1371/journal.pgph.0003852.r002

**Authors:** Miquel Vall-llosera Camps

5 Jun 2024

PGPH-D-24-00029

Institutional Trust, Conspiracy Beliefs and Covid-19 Vaccine Uptake and Hesitancy among Adults in Ghana

PLOS Global Public Health

Dear Dr. Dowou,

Thank you for submitting your manuscript to PLOS Global Public Health. Firstly, we would like to apologize for the delay in processing your manuscript. It has been exceptionally difficult to secure reviewers to evaluate your study. We have now received one completed review, which is available below. The reviewer has raised significant scientific concerns about the study that need to be addressed in a revision.

Please note that we have only been able to secure a single reviewer to assess your manuscript. We are issuing a decision on your manuscript at this point to prevent further delays in the evaluation of your manuscript. Please be aware that the editor who handles your revised manuscript might find it necessary to invite additional reviewers to assess this work once the revised manuscript is submitted. However, we will aim to proceed on the basis of this single review if possible. 

Please submit your revised manuscript by Jul 04 2024 11:59PM. If you will need more time than this to complete your revisions, please reply to this message or contact the journal office at globalpubhealth@plos.org. When you're ready to submit your revision, log on to https://www.editorialmanager.com/pgph/ and select the 'Submissions Needing Revision' folder to locate your manuscript file.

Please include the following items when submitting your revised manuscript:

- A rebuttal letter that responds to each point raised by the editor and reviewer(s). You should upload this letter as a separate file labeled 'Response to Reviewers'.
- A marked-up copy of your manuscript that highlights changes made to the original version. You should upload this as a separate file labeled 'Revised Manuscript with Track Changes'.
- An unmarked version of your revised paper without tracked changes. You should upload this as a separate file labeled 'Manuscript'.

Guidelines for resubmitting your figure files are available below the reviewer comments at the end of this letter.

We look forward to receiving your revised manuscript.

Kind regards,

Miquel Vall-llosera Camps

Staff Editor

PLOS Global Public Health

Journal Requirements:

1. Please review your reference list to ensure that it is complete and correct. If you have cited papers that have been retracted, please include the rationale for doing so in the manuscript text, or remove these references and replace them with relevant current references. Any changes to the reference list should be mentioned in the rebuttal letter that accompanies your revised manuscript. If you need to cite a retracted article, indicate the article’s retracted status in the References list and also include a citation and full reference for the retraction notice.

2. In the ethics statement in the Methods, you have specified that verbal consent was obtained. Please provide additional details regarding how this consent was documented and witnessed, and state whether this was approved by the IRB.

3. Please include a complete copy of PLOS’ questionnaire on inclusivity in global research in your revised manuscript. Our policy for research in this area aims to improve transparency in the reporting of research performed outside of researchers’ own country or community. The policy applies to researchers who have travelled to a different country to conduct research, research with Indigenous populations or their lands, and research on cultural artefacts. The questionnaire can also be requested at the journal’s discretion for any other submissions, even if these conditions are not met.  Please find more information on the policy and a link to download a blank copy of the questionnaire here: https://journals.plos.org/globalpublichealth/s/best-practices-in-research-reporting. Please upload a completed version of your questionnaire as Supporting Information when you resubmit your manuscript.

4. Please amend your detailed Financial Disclosure statement. This is published with the article. It must therefore be completed in full sentences and contain the exact wording you wish to be published.

a. State the initials, alongside each funding source, of each author to receive each grant.

b. State what role the funders took in the study. If the funders had no role in your study, please state: “The funders had no role in study design, data collection and analysis, decision to publish, or preparation of the manuscript.”

c. If any authors received a salary from any of your funders, please state which authors and which funders.

If you did not receive any funding for this study, please simply state: “The authors received no specific funding for this work.”

[Note: HTML markup is below. Please do not edit.]

Reviewers' comments:

Reviewer's Responses to Questions

**Comments to the Author**

1. Does this manuscript meet PLOS Global Public Health’s publication criteria? Is the manuscript technically sound, and do the data support the conclusions? The manuscript must describe methodologically and ethically rigorous research with conclusions that are appropriately drawn based on the data presented.

Reviewer #1: Yes

**********

2. Has the statistical analysis been performed appropriately and rigorously?

Reviewer #1: Yes

**********

3. Have the authors made all data underlying the findings in their manuscript fully available (please refer to the Data Availability Statement at the start of the manuscript PDF file)?

The PLOS Data policy requires authors to make all data underlying the findings described in their manuscript fully available without restriction, with rare exception. The data should be provided as part of the manuscript or its supporting information, or deposited to a public repository. For example, in addition to summary statistics, the data points behind means, medians and variance measures should be available. If there are restrictions on publicly sharing data—e.g. participant privacy or use of data from a third party—those must be specified.

Reviewer #1: Yes

**********

4. Is the manuscript presented in an intelligible fashion and written in standard English?

PLOS Global Public Health does not copyedit accepted manuscripts, so the language in submitted articles must be clear, correct, and unambiguous. Any typographical or grammatical errors should be corrected at revision, so please note any specific errors here.

Reviewer #1: Yes

**********

5. Review Comments to the Author

Please use the space provided to explain your answers to the questions above. You may also include additional comments for the author, including concerns about dual publication, research ethics, or publication ethics. (Please upload your review as an attachment if it exceeds 20,000 characters)

Reviewer #1: Confounding bias This study was aimed to predict which factors that responsible for the vaccine uptake and vaccine hesitancy thus the confounding bias is not a main issue in this study. Please make a clear statement if the study was analyzing predictive model or causal model. If the causal model is chosen, then the confounding variables had been adjusted in model 3.

Selection bias There are no written statement pertaining how the sample were selected from four administrative cities in Ghana. Add an explanation about how the sample were chosen to tell the reader about generalizability of the study or the study was truly aimed only for the exploration of associated factors.

Information bias The questionnaire was tested for the reliability and validity from the original questionnaire in English translated to native languages. However, there were some drawbacks regarding the result of the test (reviewer can not see the appendages) and how four research assistants managed to collect 2000+ respondents in one year. Please add the results of validity and reliability testing of the questionnaire. Also add a graphic about how the sample were collected will be helpful.

Statistics bias The statistic analysis that used in this study is okay. However, there is a confusion in table 2 and 3 regarding bivariate and multivariate logistic regression in model 1, 2, and 3. Model 1,2, and so on often used to find the most robust and parsimonious model not the different model. Therefore, naming the model with number could be misleading. You can change the model 1 to the association between vaccine acceptance and trust and so on. It is common to use adjusted OR (aOR) in multivariate logistic regression, especially in model 3.

**********

6. PLOS authors have the option to publish the peer review history of their article (what does this mean?). If published, this will include your full peer review and any attached files.

**Do you want your identity to be public for this peer review?** If you choose “no”, your identity will remain anonymous but your review may still be made public.

For information about this choice, including consent withdrawal, please see our Privacy Policy.

Reviewer #1: No

**********

[NOTE: If reviewer comments were submitted as an attachment file, they will be attached to this email and accessible via the submission site. Please log into your account, locate the manuscript record, and check for the action link "View Attachments". If this link does not appear, there are no attachment files.]

While revising your submission, please upload your figure files to the Preflight Analysis and Conversion Engine (PACE) digital diagnostic tool, https://pacev2.apexcovantage.com/. PACE helps ensure that figures meet PLOS requirements. To use PACE, you must first register as a user. Registration is free. Then, login and navigate to the UPLOAD tab, where you will find detailed instructions on how to use the tool. If you encounter any issues or have any questions when using PACE, please email PLOS at figures@plos.org. Please note that Supporting Information files do not need this step.

# Author comment: Author response to Decision Letter 0

**DOI:** 10.1371/journal.pgph.0003852.r003

10 Jun 2024

Attachment Submitted filename: Response to Editor.docx

# Peer review: Decision Letter 1

**DOI:** 10.1371/journal.pgph.0003852.r004

**Authors:** Hani Mowafi

11 Sep 2024

PGPH-D-24-00029R1

Institutional Trust, Conspiracy Beliefs and Covid-19 Vaccine Uptake and Hesitancy among Adults in Ghana

PLOS Global Public Health

Dear Dr. Dowou,

Thank you for submitting your manuscript to PLOS Global Public Health. We were able to obtain more reviewers for this revised submission, and , after careful consideration, we feel that it has merit but does not fully meet PLOS Global Public Health’s publication criteria as it currently stands. Therefore, we invite you to submit a revised version of the manuscript that addresses the points raised during the review process.

==============================

Please note that Reviewers 4 & 5 identified a few typos and errors in the text as well as a few areas for improvement in clarity.  

Reviewer 2 suggests additional citations. While the authors are welcome to consider whether this would add to the background our discussion for this study, I do not believe this addition is required to meet the publication criteria. 

==============================

Please submit your revised manuscript by Oct 11 2024 11:59PM. If you will need more time than this to complete your revisions, please reply to this message or contact the journal office at globalpubhealth@plos.org. When you're ready to submit your revision, log on to https://www.editorialmanager.com/pgph/ and select the 'Submissions Needing Revision' folder to locate your manuscript file.

Please include the following items when submitting your revised manuscript:

- A rebuttal letter that responds to each point raised by the editor and reviewer(s). You should upload this letter as a separate file labeled 'Response to Reviewers'.
- A marked-up copy of your manuscript that highlights changes made to the original version. You should upload this as a separate file labeled 'Revised Manuscript with Track Changes'.
- An unmarked version of your revised paper without tracked changes. You should upload this as a separate file labeled 'Manuscript'.

Guidelines for resubmitting your figure files are available below the reviewer comments at the end of this letter.

We look forward to receiving your revised manuscript.

Kind regards,

Sarah E. Brewer, PhD

Academic Editor

PLOS Global Public Health

Journal Requirements:

1. In the ethics statement in the Methods, you have specified that verbal consent was obtained. Please provide additional details regarding how this consent was documented and witnessed, and state whether this was approved by the IRB

2. Please include a complete copy of PLOS’ questionnaire on inclusivity in global research in your revised manuscript. Our policy for research in this area aims to improve transparency in the reporting of research performed outside of researchers’ own country or community. The policy applies to researchers who have travelled to a different country to conduct research, research with Indigenous populations or their lands, and research on cultural artefacts. The questionnaire can also be requested at the journal’s discretion for any other submissions, even if these conditions are not met.  Please find more information on the policy and a link to download a blank copy of the questionnaire here: https://journals.plos.org/globalpublichealth/s/best-practices-in-research-reporting. Please upload a completed version of your questionnaire as Supporting Information when you resubmit your manuscript.

Please review your reference list to ensure that it is complete and correct. If you have cited papers that have been retracted, please include the rationale for doing so in the manuscript text, or remove these references and replace them with relevant current references. Any changes to the reference list should be mentioned in the rebuttal letter that accompanies your revised manuscript. If you need to cite a retracted article, indicate the article’s retracted status in the References list and also include a citation and full reference for the retraction notice.

Additional Editor Comments (if provided):

[Note: HTML markup is below. Please do not edit.]

Reviewers' comments:

Reviewer's Responses to Questions

**Comments to the Author**

1. If the authors have adequately addressed your comments raised in a previous round of review and you feel that this manuscript is now acceptable for publication, you may indicate that here to bypass the “Comments to the Author” section, enter your conflict of interest statement in the “Confidential to Editor” section, and submit your "Accept" recommendation.

Reviewer #2: (No Response)

Reviewer #3: All comments have been addressed

Reviewer #4: (No Response)

Reviewer #5: All comments have been addressed

**********

2. Does this manuscript meet PLOS Global Public Health’s publication criteria? Is the manuscript technically sound, and do the data support the conclusions? The manuscript must describe methodologically and ethically rigorous research with conclusions that are appropriately drawn based on the data presented.

Reviewer #2: Yes

Reviewer #3: Yes

Reviewer #4: Yes

Reviewer #5: Yes

**********

3. Has the statistical analysis been performed appropriately and rigorously?

Reviewer #2: Yes

Reviewer #3: Yes

Reviewer #4: Yes

Reviewer #5: Yes

**********

4. Have the authors made all data underlying the findings in their manuscript fully available (please refer to the Data Availability Statement at the start of the manuscript PDF file)?

The PLOS Data policy requires authors to make all data underlying the findings described in their manuscript fully available without restriction, with rare exception. The data should be provided as part of the manuscript or its supporting information, or deposited to a public repository. For example, in addition to summary statistics, the data points behind means, medians and variance measures should be available. If there are restrictions on publicly sharing data—e.g. participant privacy or use of data from a third party—those must be specified.

Reviewer #2: (No Response)

Reviewer #3: Yes

Reviewer #4: Yes

Reviewer #5: Yes

**********

5. Is the manuscript presented in an intelligible fashion and written in standard English?

PLOS Global Public Health does not copyedit accepted manuscripts, so the language in submitted articles must be clear, correct, and unambiguous. Any typographical or grammatical errors should be corrected at revision, so please note any specific errors here.

Reviewer #2: Yes

Reviewer #3: Yes

Reviewer #4: Yes

Reviewer #5: Yes

**********

6. Review Comments to the Author

Please use the space provided to explain your answers to the questions above. You may also include additional comments for the author, including concerns about dual publication, research ethics, or publication ethics. (Please upload your review as an attachment if it exceeds 20,000 characters)

Reviewer #2: Comment to the authors on "Institutional Trust, Conspiracy Beliefs and Covid-19 Vaccine Uptake and Hesitancy among Adults in Ghana"

Background

While an attempt has been made to justify the need of this important study in light of COVID-19 public health concerns, there is still much to be done. For instance, a cursory search revealed that the following study “Morgan, A. K., Aziire, M. A., Cobbold, J., Agbobada, A. A., & Kudzawu, S. K. (2023). Hesitant or not: A cross-sectional study of socio-demographics, conspiracy theories, trust in public health information, social capital and vaccine hesitancy among older adults in Ghana. Human Vaccines & Immunotherapeutics, 19(1), 2211495” was excluded from the literature relied on. Despite the close proximity, not only in geographical or conceptual scope,. I believe a thorough literature search is needed to ground the premise of the study and also for the discussion of the findings thereof.

Study Context and Setting

The study context rather looks outdated. Spruce it up to make it current; particularly with regard to the figures, etc…

Results

Use contingency tables and chi square texts to show if there’s any form or association between vaccine uptake or otherwise and the socio-demographic factors in Table 1.

Discussion

What are the factors that account for the low vaccine uptake among those who tested positive for COVID-19?

How did the definition and categorization of the variables influence the results or findings?

Reviewer #3: The issues that were raised for review were all addressed.

Reviewer #4: This is an interesting study which focuses on potential impediments to vaccine uptake in low and middle income countries, including issues of institutional trust and potential conspiracy theories. I am reviewing for the first time a revised version of the document. Previous reviewers have focused primarily on statistical methodologies and I feel that all of these comments have been adequately addressed by the authors. The manuscript is nearly ready for publication but would benefit from selected revisions that are noted below under "SPECIFIC RECOMMENDATIONS"

Currently there are numerous published studies that assess levels and predictors of vaccine hesitancy surrounding the COVID=19 pandemic. Many of these papers express the need for expansion of the role that public health staff and institutions should play to further reduce vaccine hesitancy. However, there are often no specific recommendations as to what courses of action that the public health sector could play and what unique strengths and weaknesses do they bring. This paper in the Discussion section (Lines 390 to 404) does provide some specific recommendations. However it would be extremely helpful it they could provide relevant references for each of their recommendations. Specifically, on how to build trust in public institutions, increasing provider's capacity and ability to provide patient education, how to better use social media, and expanding information to patients on a wider range of vaccines.

SPECIFIC RECOMMENDATIONS: (I am using the revised manuscript and the accompanying Line numbering)

ABSTRACT:

Line 34: I would suggest revising the sentence, "Vaccine hesitancy is considered by the World Health Organization as one of the ten threats to global health. "

Line 36: The authors use the terms, "vaccine hesitancy" and "vaccine hesitance" interchangeably. Is there a distinction between these two terms??

MAIN MANUSCRIPT:

Line 64-65: While I agree that COVID-19 is no longer officially considered a public health emergency of international concern, it remains a public health priority for WHO.

Line 111. Would suggest writing out in full.." Sub Saharan Africa"

Lines 325-326. This sentence is incomplete. "This is particularly critical considering the evolving nature of the various........???????" Please complete the sentence.

Reviewer #5: The following need to be incorporated before final approval of the manuscript:

1. Line 71 - the 14.4 billion should be replaced by 14.4 million

2. Line 77-78 : Acceptance / use - need to add the dimension of effective quality chain and concerns about the quality at least in the description for the COVID vaccine, which had very strict temparature requirments - that was not easy to maintain tropical Ghana.

3. The authoris need to make a specific comment upfront about "Institutional Trust" as represented by the comments on people in the instituitons. For example, the authors did not ask, do you trust the health centre or do your trust dispensary or hospital. Potentially, there is a difference between institutions, as opposed to individuals in the institutions. It is best to qualify it upfront both in the Methods and in the discussion sections.

**********

7. PLOS authors have the option to publish the peer review history of their article (what does this mean?). If published, this will include your full peer review and any attached files.

**Do you want your identity to be public for this peer review?** If you choose “no”, your identity will remain anonymous but your review may still be made public.

For information about this choice, including consent withdrawal, please see our Privacy Policy.

Reviewer #2: **Yes: **Anthony Kwame Morgan

Reviewer #3: **Yes: **Samoel Ashimosi Khamadi

Reviewer #4: **Yes: **Paul R De Lay, MD, DTM&H (Lond)

Reviewer #5: **Yes: **Lakshmi N Balaji

**********

[NOTE: If reviewer comments were submitted as an attachment file, they will be attached to this email and accessible via the submission site. Please log into your account, locate the manuscript record, and check for the action link "View Attachments". If this link does not appear, there are no attachment files.]

While revising your submission, please upload your figure files to the Preflight Analysis and Conversion Engine (PACE) digital diagnostic tool, https://pacev2.apexcovantage.com/. PACE helps ensure that figures meet PLOS requirements. To use PACE, you must first register as a user. Registration is free. Then, login and navigate to the UPLOAD tab, where you will find detailed instructions on how to use the tool. If you encounter any issues or have any questions when using PACE, please email PLOS at figures@plos.org. Please note that Supporting Information files do not need this step.

# Author comment: Author response to Decision Letter 1

**DOI:** 10.1371/journal.pgph.0003852.r005

23 Sep 2024

Attachment Submitted filename: response_COVID.pdf

# Editor report: Decision Letter 2

**DOI:** 10.1371/journal.pgph.0003852.r006

**Authors:** Sarah E. Brewer

30 Sep 2024

Institutional Trust, Conspiracy Beliefs and Covid-19 Vaccine Uptake and Hesitancy among Adults in Ghana

PGPH-D-24-00029R2

Dear Mr Dowou,

We are pleased to inform you that your manuscript 'Institutional Trust, Conspiracy Beliefs and Covid-19 Vaccine Uptake and Hesitancy among Adults in Ghana' has been provisionally accepted for publication in PLOS Global Public Health.

Before your manuscript can be formally accepted you will need to complete some formatting changes, which you will receive in a follow up email. A member of our team will be in touch with a set of requests.

Please note that your manuscript will not be scheduled for publication until you have made the required changes, so a swift response is appreciated.

IMPORTANT: The editorial review process is now complete. PLOS will only permit corrections to spelling, formatting or significant scientific errors from this point onwards. Requests for major changes, or any which affect the scientific understanding of your work, will cause delays to the publication date of your manuscript.

If your institution or institutions have a press office, please notify them about your upcoming paper to help maximize its impact. If they'll be preparing press materials, please inform our press team as soon as possible -- no later than 48 hours after receiving the formal acceptance. Your manuscript will remain under strict press embargo until 2 pm Eastern Time on the date of publication. For more information, please contact globalpubhealth@plos.org.

Thank you again for supporting Open Access publishing; we are looking forward to publishing your work in PLOS Global Public Health.

Best regards,

Sarah E. Brewer, PhD

Academic Editor

PLOS Global Public Health

***********************************************************

Reviewer Comments (if any, and for reference):
